# CT-guided minimally-invasive penile fracture repair

**DOI:** 10.1590/S1677-5538.IBJU.2018.0525

**Published:** 2019

**Authors:** Cui Yan, Bing-xue Liang, Hai-bin Huang, Bi-rong Liang, Zheng Zhou, Ling-jun Wang, Zhong-qi Yang, Shao-xiang Xian

**Affiliations:** 1 Department of Traditional Chinese Medicine, the First Affiliated Hospital of Guangzhou University of Chinese Medicine, Guangzhou 510405, China

**Keywords:** Penis, Urology, Sutureless Surgical Procedures, Tomography, X-Ray Computed

## Abstract

We present the case of a 28 year old patient with an incomplete tear of the tunica albuginea occurred after having sexual intercourse in the female superior position. The diagnostic assessment was performed first clinically, then with CT, owing to its high resolution, allowed to exactly detect the tear location leading to precise preoperative planning. After adequate diagnosis through imaging and proper planning, the patient was performed a selective minimally invasive surgical approach to repair the lesion. The patient had good erection with no angular deformity or plaque formation after a 3-month follow-up.

## INTRODUCTION

Penile fracture is a rare clinical entity that represents a urologic emergency, defined as unilateral or bilateral rupture of tunica albuginea of corpora cavernosa. The most common mechanism of injury is non-physiologic bending of an erect penis during sexual intercourse (1). The patient experiences a sudden cracking sensation and severe pain. The erection resolves immediately, and subsequently penis will be severely edematous, congestion, and stays angulated toward the unaffected side resulting in a characteristic appearance resembling an eggplant.

Although the clinical diagnosis is often straightforward, preoperative medical imaging can show any associated complication (2–4). Imaging modalities such as ultrasound, cavernosography, retrograde urethrography, magnetic resonance imaging (MRI), and computed tomography (CT) may be of helping determining the exact site of tunica lesion preoperatively and customizing the surgical repair (5, 6).

Ultrasound is most commonly used for early diagnosis (7). However, the low sensitivity for small tear of the tunica albuginea and the high operator dependence are major limitations. Retrograde urethrography and cavernosography are rarely performed because of invasive operation. Although MRI may represent the extension and the exact tear of the tunica albuginea, it is not convenient for emergency patients. CT can be performed at emergency and plays a key role for treatment planning. CT high resolution images permit to distinguish a real tunica albuginea rupture, from a false rupture due to a dorsal vein tear or an isolated intracavernous hematoma.

To our knowledge, ultrasound and MRI findings of penile fracture have been previously reported in the literature. However, limited information is available concerning CT findings of penile fracture. As a result, we present a case report on CT-guided minimally-invasive penile fracture repair.

## Case report

**Figure 1:**
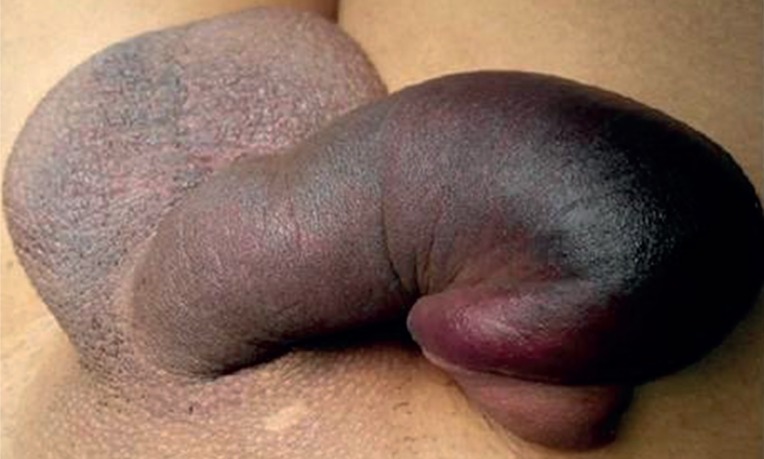
Twenty-eight year-old man with penile fracture. Clinical photograph showed a markedly swollen, purple and painful penis resembling an eggplant.

**Figure 2:**
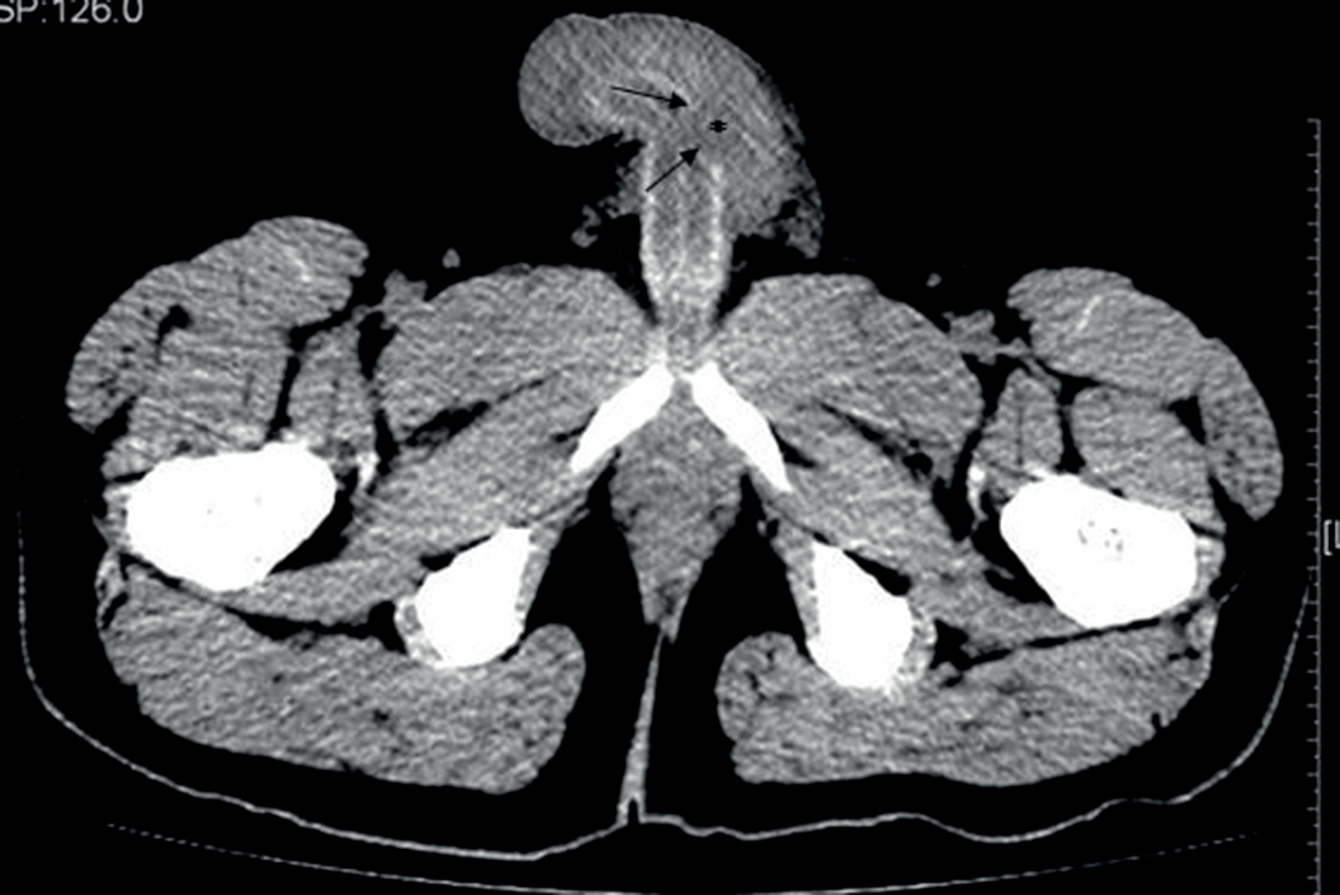
Twenty-eight - old man with penile fracture. Axial CT image obviously demonstrated unilateral 0.8cm x 1.4cm area of tear of the left lateral tunia albuginea with adjacent hematoma. Arrows indicate the free edges of tunica defect and asterisks show the associated hematoma.

A 28-year-old man presented to our medical center with pain and asymmetric penile swelling, following a penile injury sustained 1 hour previously. He described having sexual intercourse in the female superior position when, on attempting penetration, his penis bent sharply against his partner's thigh. He noticed a ‘snap’ in the left side of his penis, associated with severe pain, discoloration and deformation. The penis then began to swell and marked bruising developed along the shaft, eventually tracking down into the scrotum. He was able to void normally and had not noticed blood in his urine. On physical examination, the patient had a grossly deformed penis with swelling and deviation to the right (Figure-1). There was no microscopic hematuria on dipstick urinalysis and urethral imaging was, therefore, not performed. Axial CT image demonstrated unilateral 0.8cm × 1.4cm area of tear of the left lateral tunica albuginea with adjacent hematoma. The penile shaft deviates to the right side. The imaging findings were confirmed at surgery (Figure-2).

**Figure 3:**
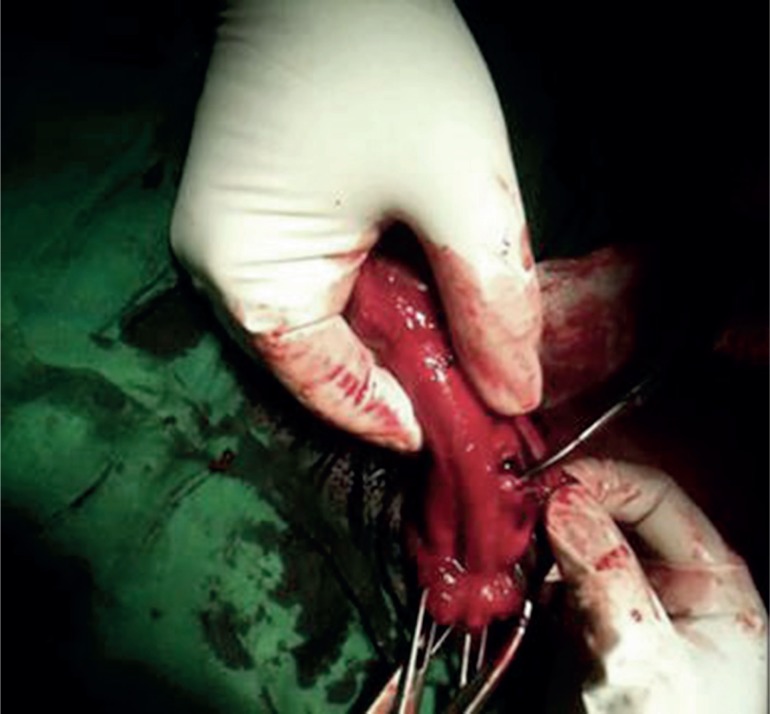
The rent in tunica albuginia and corpora cavernosum. The haematoma within the tunica albuginea was evacuated and the corporal tear was identified.

The patient was transferred to the operating room where, under general anesthesia, a defect in the middle of the left corpus cavernosum could be palpated by rolling a finger over the hematoma. He was treated by surgical exploration, evacuation of the haematoma and repair of the ruptured tunica albuginea using absorbable sutures (Figure-3). The patient had good erection with no angular deformity or plaque formation after a 3-month follow-up.

## DISCUSSION

The underlying pathology of penile fracture is rupture of the corpus cavernosum of an erect penis (8, 9). During erection, the tunica albuginea is stretched / thinned, and if put under strain by manipulation, the firmly engorged corpus breaks through, leading to penile fracture.

It is difficult to estimate the incidence of penile fractures: a review of 1.600 cases reported in the literature, showed a higher prevalence in Asian and Middle-Eastern countries. Vigorous sexual intercourse is considered to be the main cause of penile fractures in the Western world. Other causes are the following: non-physiologic penile bending, rolling over in bed with an erect penis and sport injuries with flaccid penis. In Middle Eastern countries the most frequent reported cause of penile fracture is a self-inflicted practice called Taghaandan which consists of snapping and bending the erect penis to achieve a rapid detumescence (10).

There are several penile fracture reports which are about the surgery procedure. Surgical treatment must be planned as soon as possible to avoid postoperative erectile dysfunction (11, 12). However, limited information is available concerning the CT findings of penile fracture. CT may precisely demonstrate the presence, location, and extent of the tunical tear, which manifests as discontinuity of the tunica albuginea (13). As a result, CT could save the surgical exploration time. So it is necessary for a preoperative evaluation with the imaging technique, though penile fracture can be diagnosed from the history and physical examination.

In this case, penile fracture occurred as intercourse in the female superior position when, on attempting penetration, the patient's penis bent sharply against his partner's thigh. In our patient, after detecting the location of the rupture with CT, he was treated by surgical exploration, evacuation of the haematoma and repair of the ruptured tunica albuginea using absorbable sutures, avoiding unnecessary circumcision, reducing the risk for necrotic and sensitivity complications.

To our knowledge, CT-guided minimally-invasive penile fracture repair has not been previously reported in the literature. In such case, CT would facilitate prompt diagnosis and early treatment, maximizing the chance of a good long-term clinical outcome. So, penile fracture is a condition that requires accurate diagnosis for treatment planning. Though MRI is a radiation free alternative and the best radiological tool in terms of soft tissue resolutions compared to CT, CT imaging provides the key information to offer the best treatment minimizing risks of complications at emergency.
